# Characteristics of test anxiety among medical students and congruence of strategies to address it

**DOI:** 10.3402/meo.v19.25211

**Published:** 2014-08-13

**Authors:** John Encandela, Crystal Gibson, Nancy Angoff, Gary Leydon, Michael Green

**Affiliations:** 1 Teaching and Learning Center, Yale School of Medicine, New Haven, CT, USA; 2 Department of Psychiatry, Yale School of Medicine, New Haven, CT, USA; 3 Office of Student Affairs, Yale School of Medicine, New Haven, CT, USA; 4 Department of Internal Medicine, Yale School of Medicine, New Haven, CT, USA

**Keywords:** test anxiety, high-stakes exams, test-taking, medical students

## Abstract

**Introduction:**

Medical students may experience test anxiety associated with ‘high stakes’ exams, such as Step 1 of the United States Medical Licensing Examination.

**Methods:**

We collected qualitative responses about test anxiety at three points in time from 93 second-year medical students engaged in studying for and taking Step 1.

**Results:**

*Causes* of test anxiety as reported by students were related to negative self-talk during preparation for the exam. *Effects* of anxiety had to do with emotional well-being, cognitive functioning, and physical well-being. Strategies included socializing with others and a variety of cognitive and physical approaches. Comparison of individuals’ strategies with causes and effects showed some congruence, but substantial incongruence between the types of strategies chosen and the reported causes and effects of test anxiety.

**Discussion:**

Students’ adoption of a ‘menu’ of strategies rather than one or two carefully selected strategies suggest inefficiencies that might be addressed by interventions, such as advisor-directed conversations with students and incorporating student self-assessment and strategies for managing anxiety within courses on test-taking. Such interventions are in need of further study. An annotated list of evidence-based strategies would be helpful to students and educators. Most important, test anxiety should be viewed by medical educators as a ‘real’ experience, and students would benefit from educator support.

Step 1 of the United States Medical Licensing Examination (USMLE) is considered to be ‘high stakes’ by many medical students since passing it may be required to graduate from medical school, and it is widely used by residency program directors as a measure of performance when choosing new residents (1–3). Because of this high-stakes status, students often experience test anxiety while preparing for and taking Step 1 (3, 4). Medical school curricula in the U.S. seldom *formally* address anxiety associated with the Step 1 or other standardized examinations associated with medical training. Consequently, many students are left to their own devices to prepare for these exams and address associated anxiety (3).^1^

At our medical school, we piloted a test-preparation course and studied its effects on test anxiety among second-year students preparing for Step 1. Quantitative results are presented elsewhere (5). We used the opportunity to also collect narrative information from the entire cohort of second-year students regarding their past and current experiences with test anxiety. Their perceptions were very informative about the nature and extent of anxiety they encountered. Often, we as faculty perceive our students to be academically astute and self-assured; therefore, the amount of anxiety expressed was surprising to us. In this paper, we would like to share some of the perceptions of these students and suggest implications for further intervention and research.

## Methods

Qualitative data were gathered in conjunction with administration of the Westside Test Anxiety Scale that was used in the aforementioned pilot (6). We collected information at three points: before a formal study period for Step 1; during the study period; and after the vast majority of students had taken the exam. Ninety-three out of 101 second-year students voluntarily participated. Questions that prompted narrative answers involved experiences that students have had with test anxiety in the past as well as in preparation for Step 1; characteristics of this anxiety; and strategies that respondents may have used successfully to address test anxiety. We processed this information with standard, thematic analysis of qualitative data.

Themes and findings were organized by three large categories: causes of, effects of, and strategies for, managing anxiety. We compared students’ descriptions of strategies with these same individuals’ explanations of causes and effects of anxiety to arrive at a sense of whether strategies seemed to correspond with causes and effects.

## Results

In students’ narratives, they described *causes* of test anxiety to be related to cognitive activities, or ‘self-talk’, that they performed while studying for and taking exams like Step 1. Self-talk focused on the perception of the ‘high stakes’ of exams; prior academic performance and how students perceived this to influence new exam-taking; perceived time constraints for study; and academic comparison with peers. A quote from one respondent represents the type of self-talk described by many:

My main worry is how much this exam is supposed to mean and how much I'm doing compared with those around me. I realize I should not be comparing myself or listening to the worries of my peers, but it's hard to shut that out—especially when people very close to me (and who I believe to be much smarter than me) have been studying head over heals for the exam for a long time. I'm definitely afraid I won't have studied enough, and have not done enough these past two years to prepare me for this test.

*Effects* of test anxiety had to do with stressors on emotional or mental well-being, cognitive functions, and physical well-being. One respondent described the emotional ‘ups-and-downs’ that result from anxiety when preparing for exams:

When it comes close to exam time I cram since I've procrastinated studying and preparing for the exam. The stress isn't crippling but it will interfere where I'll frantically swipe through pages of a book hoping something sticks. It depends on the day, too. I'll cyclically go through stages where I feel confident, intimidated, uncertain, anxious, irritable, frantic, then relaxed again.

Students also described the effects of anxiety on cognition. Specifically, respondents explained that the ability to concentrate and the ability to remember material have been both affected by anxious thoughts. One student said that, due to anxiety during the time of test preparation, ‘I couldn't focus on the material and felt like I didn't know what I should do with the time I had left [to study]’.

Another explained that during test-taking itself, ‘my nervousness made it hard for me to remember specific details or equations that would have helped me answer questions’.

In addition to effects on cognition and emotions, students claimed that test anxiety took a toll on physical well-being. A number of students reported difficulty in sleeping during exam preparation and especially the night before an exam. Anxiety also had effects on appetite and eating habits. Some explained that they had stopped eating regularly during test preparation, and others described eating unhealthy snacks in an attempt to ease anxiety, which in turn, negatively affected the way they felt physically.

Students also reported adopting a number of *strategies* to manage anxiety, the most common of which involved deliberate attempts to socialize with friends and family throughout test preparation and up to the time of the exam. Students also noted a number of cognitive and physical strategies. Examples of cognitive strategies were the practice of mindfulness and deliberate re-orientation of thinking; redirecting ‘self-talk’ in a way that placed importance of the exam in context with the rest of life; and focusing on prayer, meditation, yoga, and spirituality. Physical strategies included managing sleep and eating patterns, focusing on healthy eating, maintaining well-planned study breaks, balancing study with fun and physical activity, and maintaining an exercise regime.

Individuals’ *descriptions of strategies were compared with perceived causes and effects* of anxiety. In some cases, strategies seemed to match causes and effects (for instance, negative self-talk and effects on emotions may have been addressed by seeking help from a counselor). However, in *most* cases, individuals listed a ‘menu’ of strategies they had attempted—some of these strategies seemed to match and some seemed to not correspond with these causes and effects. This made us wonder if many students expended too much energy and resources on managing anxiety through preparation up to the day of the exam, during a period when time and energy are typically ‘at a premium’.

## Discussion

Students’ explanations of their experiences with test anxiety and strategies they have tried for managing it suggest possible inefficiencies. This may have to do with the fact that students are often left to their own devices rather than having resources leading to evidence-based approaches to curb anxiety. If the longer ‘menus’ of strategies could be replaced by one or two approaches per student that address the core causes and most debilitating effects of test anxiety, students may find both a level of relief and more efficient time to prepare for high-stakes exams.

This observation has several implications for possible interventions and study. First, advisors to these students could be a vital resource by assisting in the assessment of test anxiety and introducing students to strategies that best address the distinct causes and effects. When meeting with students, advisors may have the opportunity to draw on students’ own perceptions of reasons for and effects of anxiety (which are important as subjective appraisals), but also may use objective measures. The Westside Test Anxiety Scale that was part of the quantitative portion of our study is one possible tool since it is composed of two subscales: one measuring ‘worry’, which is associated with ‘catastrophizing’ thinking and self-talk; the other measuring incapacity, specifically memory loss and poor cognitive processing (6). Attention to ways in which students score on these sub-scales lend to specifying causes and effects of test anxiety; these results coupled with students’ own appraisals would provide useful information for, then, advising on strategies.

Additionally, having students self-assess and, then, strategize approaches for managing anxiety might be incorporated in future test-preparation courses. Students could complete assessment inventories individually, and as a group learn about appropriate strategies for their particular manifestations of test anxiety. Doing this in the context of a course might result in students’ mutual support in their encounters with anxiety. On the other hand, they may prefer to keep their anxieties private from peers. In any case, such an approach might be worth testing for its acceptance and effectiveness.

These possible interventions call for knowledge of evidence-based strategies that have worked in addressing test anxiety. A concerted effort to compile an annotated list of strategies would be useful to both students and educators.

Last, the narrative perceptions of our students has made us aware of the intensity and commonly experienced nature of anxiety. Any ‘empty advice’ we might have given ‘not to worry, you will do fine’ will certainly be replaced with more thoughtful discussions on the causes, effects, and management of their test anxiety in the future.

## Footnotes

1 An exception is a five-part, process-oriented preparation implemented at one U.S. medical school that had the effect of reducing anxiety associated with Step 1 (3).
